# Supplementary material for: Multi-faceted immunomodulatory and tissue-tropic clinical bacterial isolate potentiates prostate cancer immunotherapy
Source: Nat Commun. 2018 Apr 23;9:1591. doi: 10.1038/s41467-018-03900-x (PMC5913311; doi:10.1038/s41467-018-03900-x)
Supplement: Supplementary file 1 — Supplementary Information [file 41467_2018_3900_MOESM1_ESM.pdf]

# Multi-faceted immunomodulatory and tissue-tropic clinical bacterial isolate potentiates prostate cancer immunotherapy

Anker et al.

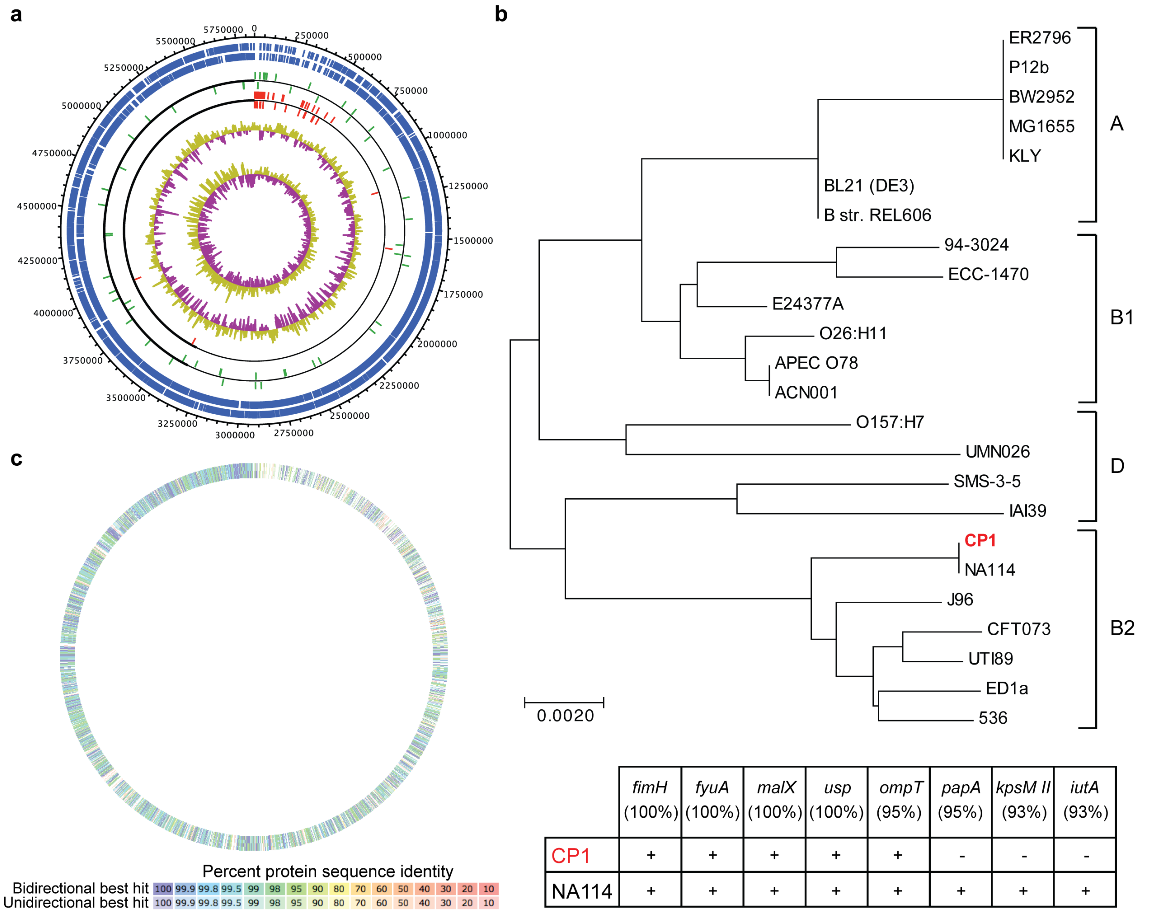


| | *fimH* (100%) | *fyuA* (100%) | *malX* (100%) | *usp* (100%) | *ompT* (95%) | *papA* (95%) | *kpsM II* (93%) | *iutA* (93%) |
|---|---|---|---|---|---|---|---|---|
| CP1 | + | + | + | + | + | - | - | - |
| NA114 | + | + | + | + | + | + | + | + |

**Supplementary Figure 1. Whole genome sequencing of CP1.** (**a**) The sequenced CP1 genome was visualized with Artemis DNAPlotter. Tracks from outermost to innermost: forward coding sequence (CDS), reverse CDS, forward tRNA, reverse tRNA, forward rRNA, reverse rRNA, GC plot, GC skew. (**b**) Phylogenetic tree of CP1 with reference *E. coli* strains using the Maximum Likelihood method with concatenated MLST sequences, constructed with MEGA7. Branch lengths were measured in the number of substitutions per site. Phylogenetic groups A, B1, D, or B2 are indicated. Table depicts the presence or absence of ST131 consensus virulence factor genes (with overall ST131 population prevalence indicated) in the CP1 or NA114 genomes. (**c**) Sequence comparison of the CP1 genome with the MG1655 genome, performed with RAST.

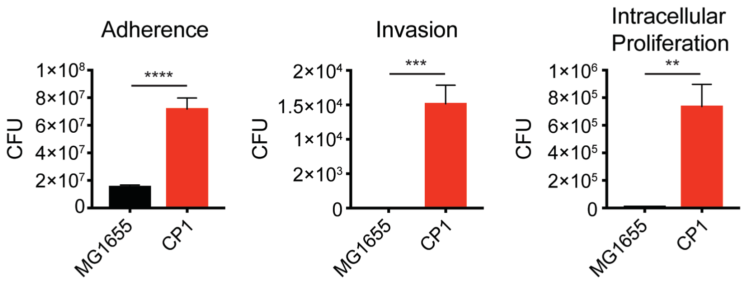


**Supplementary Figure 2. CP1 adheres to, invades, and intracellularly proliferates within prostate cancer cells.** (**a**) Gentamicin protection assay with CP1 and MG1655 with Myc-CaP cells *in vitro*, performed in sextuplicates, plated in serial dilutions. Data represented as mean ± S.E.M. Statistical significance was determined by Student's *t*-test. ** $P$<0.01, *** $P$<0.001, **** $P$<0.0001.

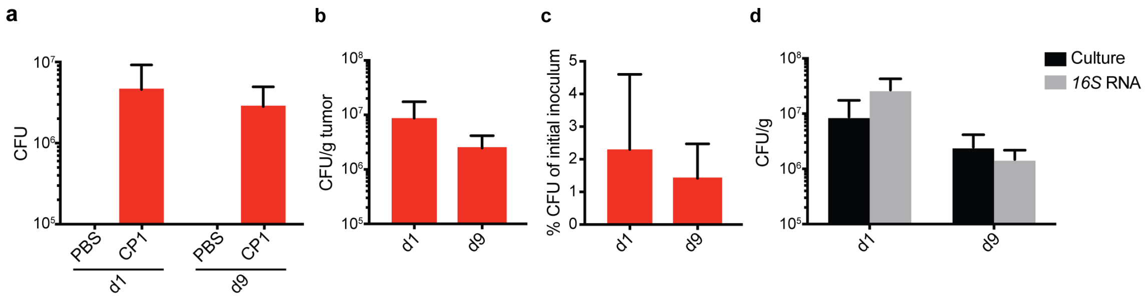


**Supplementary Figure 3. Intra-tumoral CP1 is culturable and colonization levels remain constant over time.** (**a**) Total bacterial colonization, (**b**) bacterial colonization normalized to tumor weight, and (**c**) bacterial colonization as a percentage of the original $2\times10^8$ CP1 inoculum, performed on day 1 (d1) and day 9 (d9) after intra-urethral PBS or CP1 administration to orthotopic Myc-CaP prostate tumor-bearing mice. (**d**) Bacterial colonization/g tumor as determined by both cultured tumor tissue and *16S* RT-PCR calibrated to CP1 counts on day 1 and day 9 after CP1 administration. Mice $n$ = 4-5/group, tissue cultures plated in serial dilutions, technical duplicates, RT-PCR performed in technical duplicates. Data represented as mean ± S.E.M. Statistical significance was determined by (**a-c**) two-tailed Student's $t$-test, (**d**) two-way ANOVA.

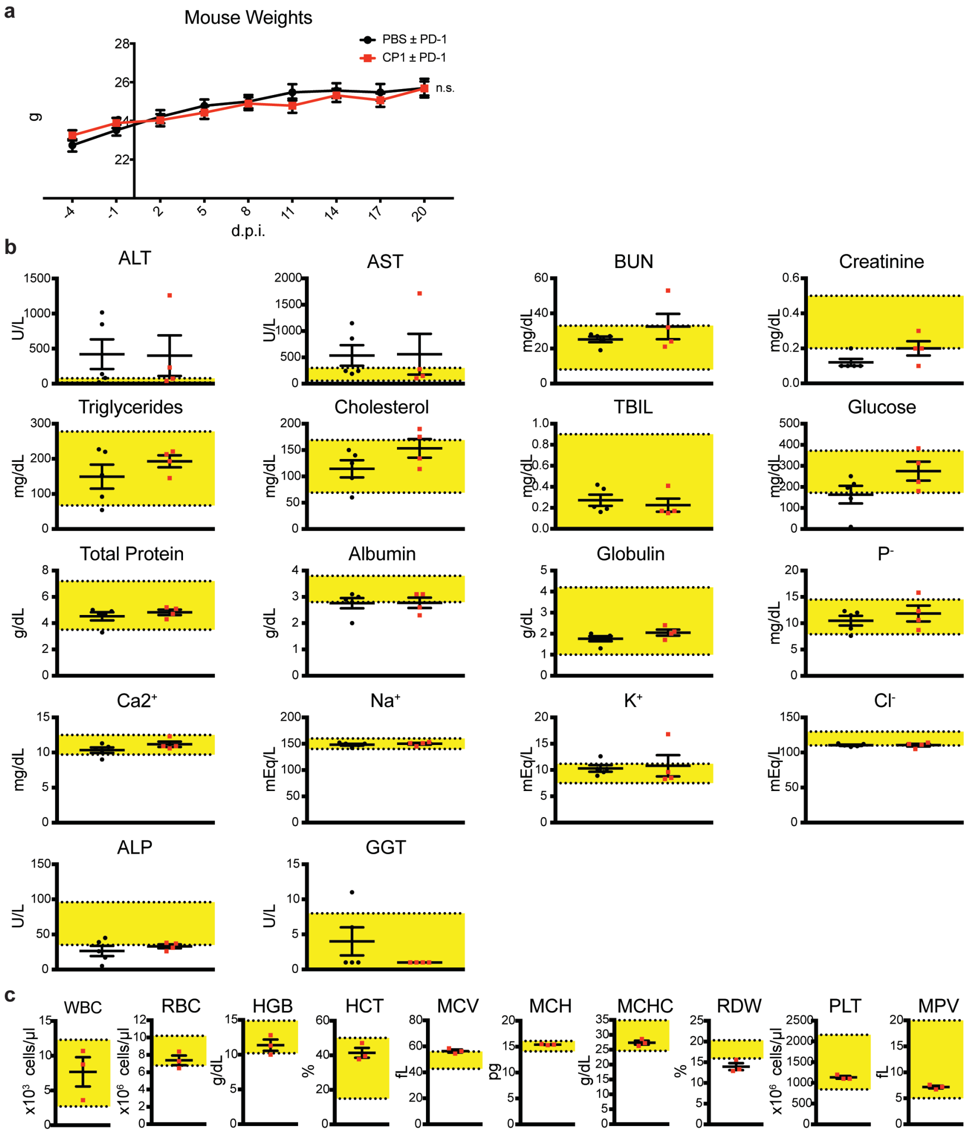


**Supplementary Figure 4. CP1 does not cause any systemic toxicities.** (**a**) Weights of PBS and CP1 administered mice (± anti-PD-1 antibody), plotted as days post-infection (d.p.i.), n.s. = not significant, *n* = 23-28 mice/experimental group. (**b**) Chemistry laboratory values of PBS and CP1 administered mice, yellow indicating the normal murine range (ALT = alanine aminotransferase, AST = aspartate aminotransferase, BUN = blood urea nitrogen, TBIL = total bilirubin, $P^-$ = phosphorous, $Ca^{2+}$ = calcium, $Na^+$ = sodium, $K^+$ = potassium, $Cl^-$ = chloride, ALP = alkaline phosphatase, GGT = gamma glutamyl transferase), *n* = 4-5 mice/experimental group. (**c**) Complete blood count (CBC) values of CP1 administered mice, yellow indicating the normal murine range (WBC = white blood cell, RBC = red blood cell, HBG = hemoglobin, HCT = hematocrit, MCV = mean corpuscular volume, MCH = mean corpuscular hemoglobin, MCHC = mean corpuscular hemoglobin concentration, RDW = RBC distribution width, PLT = platelet count, MPV = mean platelet volume), *n* = 3 mice. Data represented as mean ± S.E.M. Statistical significance was determined by (**a**) two-way ANOVA, (**b**) two-tailed Student's *t*-test.

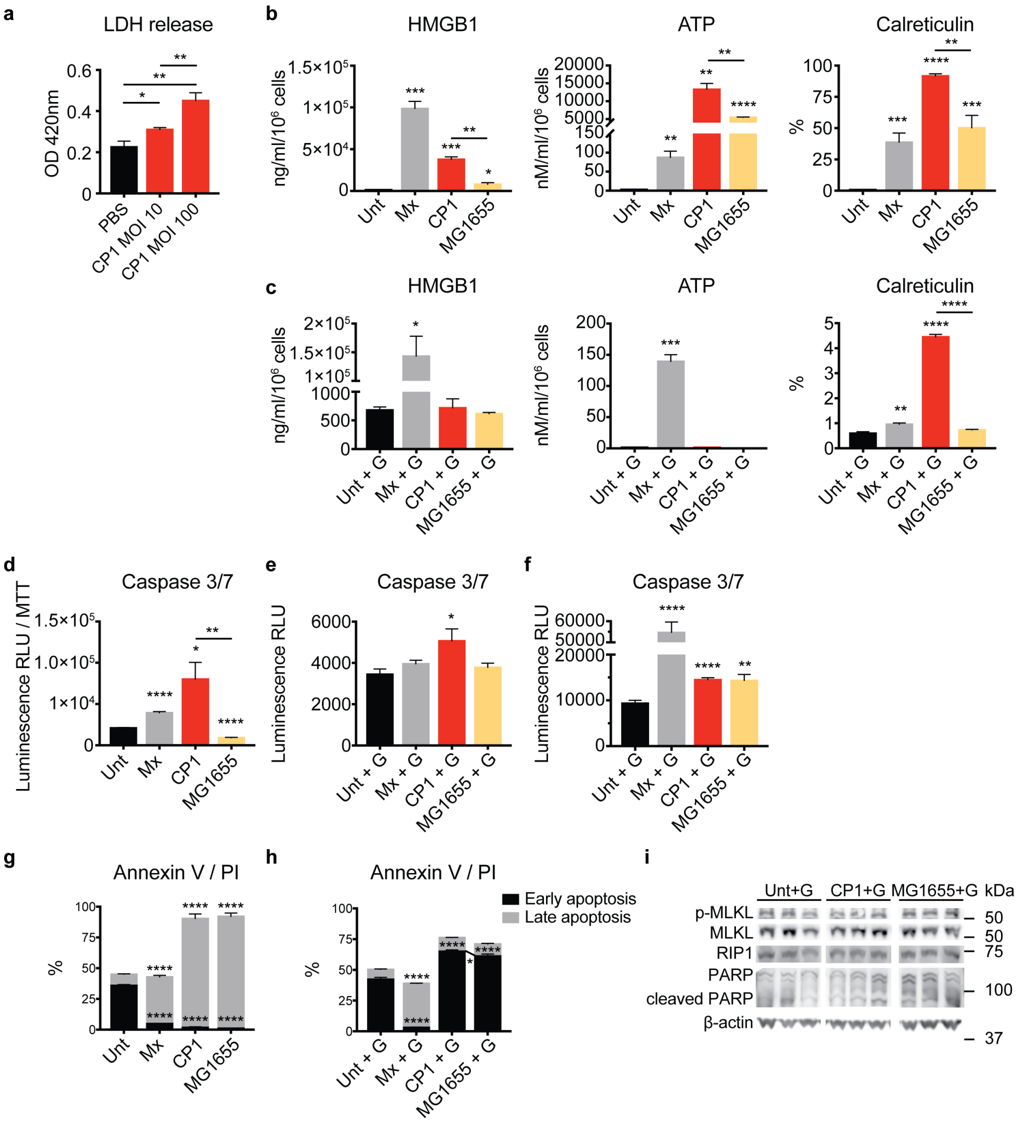


**Supplementary Figure 5. CP1 induces ICD and select cell death markers, with and without gentamicin, and to a greater degree than MG1655.** (**a**) LDH level, as a measure of cell death, from CP1 and Myc-CaP co-culture, performed in triplicates. (**b-i**) Myc-CaP cells were co-cultured with mitoxantrone (Mx), CP1 (MOI 1), or MG1655 (MOI 1) (**b, d, g**) in normal media or (**c, e, f, h, i**) with gentamicin (+ G) added after 2 hours. (**b, c**) ICD was measured via HMGB1 (ELISA, 72 hours), ATP (luminescence assay, 72 hours), and calreticulin (flow cytometry, 24 or 72 hours), performed in biological triplicates, technical duplicates. (**d-f**) Caspase 3/7 activity (luminescence assay, reported in relative light units [RLU]) was measured at (**d, e**) 6 hours or (**f**) 24 hours, (**d**) normalized to cell count (MTT assay), performed in sextuplicates. (**g, h**) Early stage apoptosis (Annexin $V^+$ $PI^-$) and late stage apoptosis (Annexin $V^+$ $PI^+$) were determined by flow cytometry after 24 hours, performed in triplicates. (**i**) Western blot analysis of phosphorylated and total MLKL, RIP1, full length and cleaved PARP, and β-actin after 24 hours, performed in triplicates. Data represented as mean ± S.E.M. Statistical significance was determined by two-tailed Student's *t*-test (each group compared to Unt, and CP1 compared to MG1655). * $P<0.05$, ** $P<0.01$, *** $P<0.001$, **** $P<0.0001$.

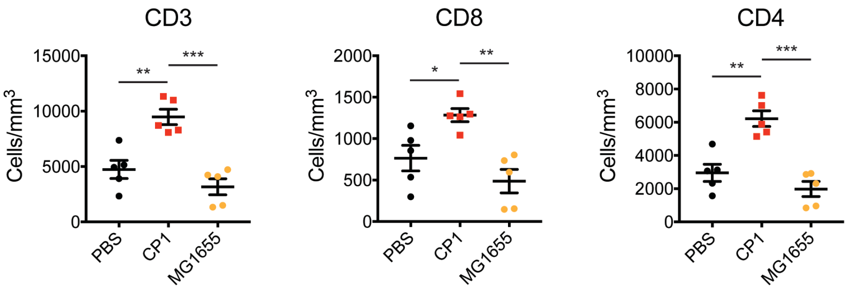


**Supplementary Figure 6. Intra-urethrally administered MG1655 does not increase prostatic TILs.** Flow cytometry analysis of orthotopic Myc-CaP tumors 9 days after intra-urethral administration of PBS, CP1, or MG1655, displayed as cell counts normalized to tumor volume. Mice $n$ = 5/group. Data represented as mean ± S.E.M. Statistical significance was determined by two-tailed Student's $t$-test. * $P<0.05$, ** $P<0.01$, *** $P<0.001$.

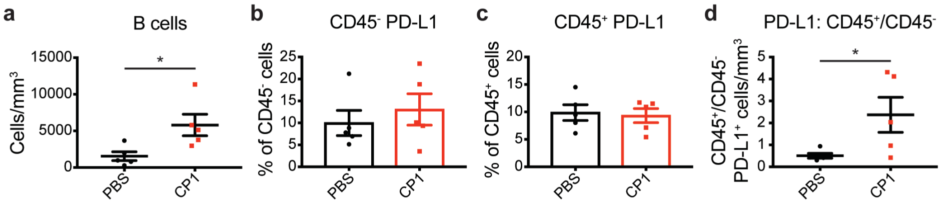


**Supplementary Figure 7. CP1 increases B cells and does not increase PD-L1 expression.** Flow cytometry analysis of (**a**) B cells, and PD-L1 on (**b**) $CD45^-$ and (**c**) $CD45^+$ intra-tumoral cells, and (**d**) the ratio of $CD45^+PD\text{-}L1^+/CD45^-PD\text{-}L1^+$ cell densities. $n$ = 4-5 mice/experimental group, performed in 2 independent experiments. Data represented as mean ± S.E.M. as cell counts normalized to tumor volume (scatter plots) or percentages of parent gate (scatter boxed plots). Statistical significance was determined by two-tailed Student's $t$-test. * $P$<0.05.

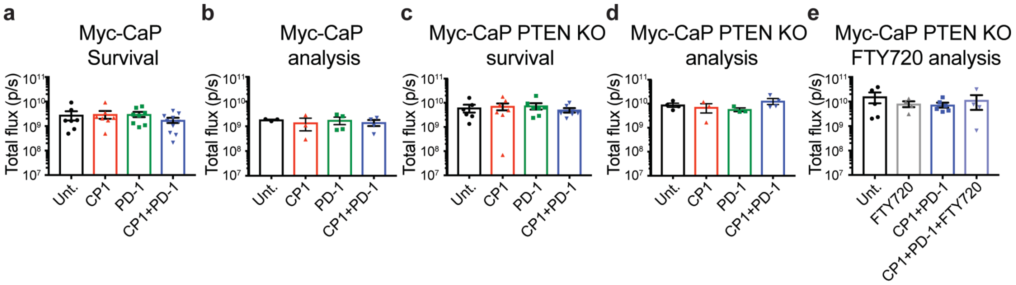


**Supplementary Figure 8. Normalization of orthotopic pre-treatment tumor burden in all *in vivo* experiments.** Pre-treatment tumor IVIS quantification of Unt., CP1, anti-PD-1 antibody, and combination CP1 and anti-PD-1 antibody treated mice in (**a**) Myc-CaP survival (Figure 4a,b, Supplementary Figure 9, Supplementary Figure 10a), (**b**) Myc-CaP analysis (Figure 4b-e), (**c**) Myc-CaP PTEN KO survival (Figure 5e), (**d**) Myc-CaP PTEN KO analysis (Figure 5f, Figure 6, Supplementary Figure 10b, Supplementary Figure 12), and (**e**) of Unt., FTY720, combination CP1 and anti-PD-1 antibody, and combination CP1 and anti-PD-1 antibody and FTY720 treated mice (Figure 7). Data represented as mean ± S.E.M. Statistical significance was determined by one-way ANOVA.

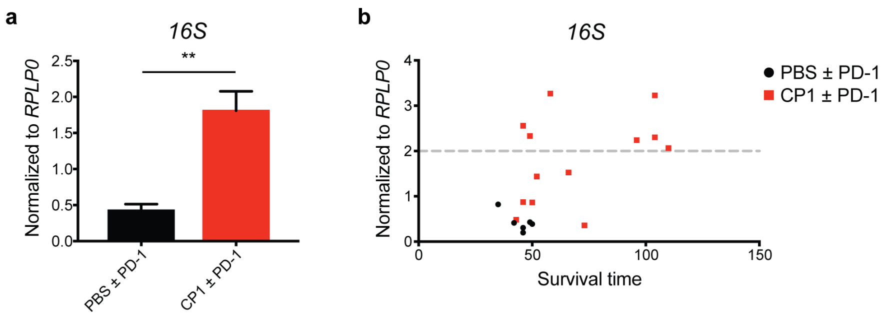


**Supplementary Figure 9. CP1 load is linked to treatment efficacy.** *16S* qRT-PCR of Myc-CaP survival mice tumors (**a**) at their endpoints and (**b**) plotted over time after tumor injection, dotted line indicates cutoff for high CP1. Data represented as mean ± S.E.M. Statistical significance was determined by two-tailed Student's *t*-test. ** $P$<0.01.

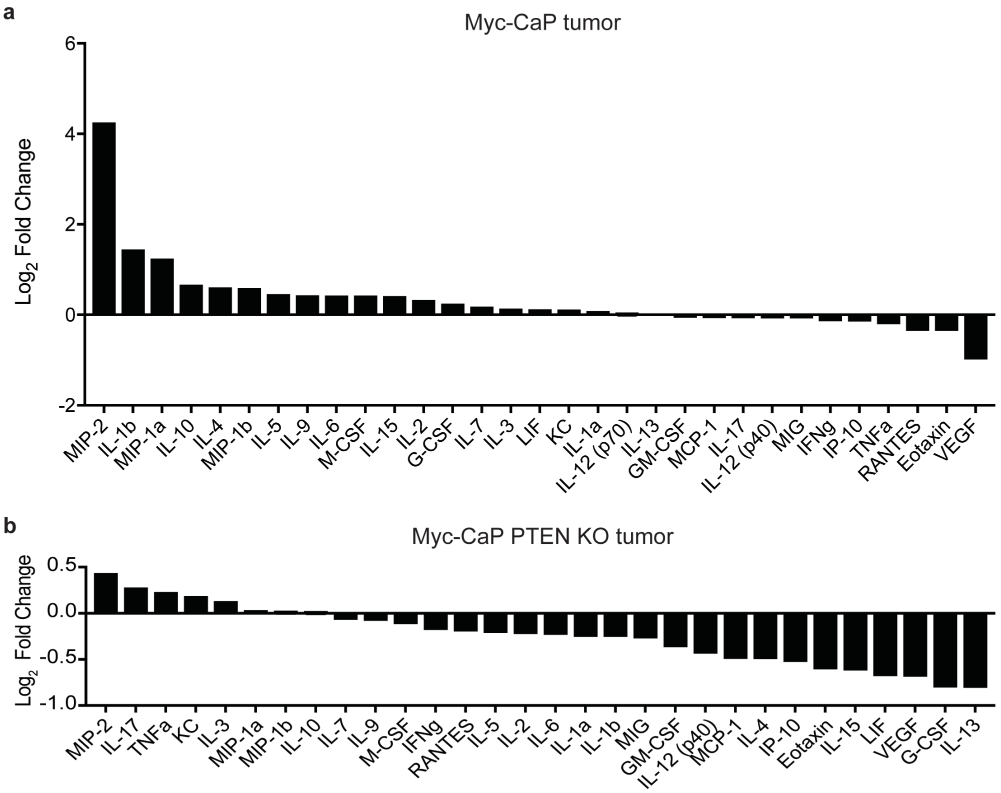


**Supplementary Figure 10. CP1 decreases intra-tumoral VEGF, increases pro-inflammatory cytokines and chemokines.** Multiplex cytokine and chemokine array from (**a**) Myc-CaP survival tumors, performed with $n$ = 11-12 mice/experimental group, and from (**b**) Myc-CaP PTEN KO tumors, performed with $n$ = 5-6 mice/experimental group and technical duplicates. Data represented as $\log_2$ fold change with and without CP1 administration.

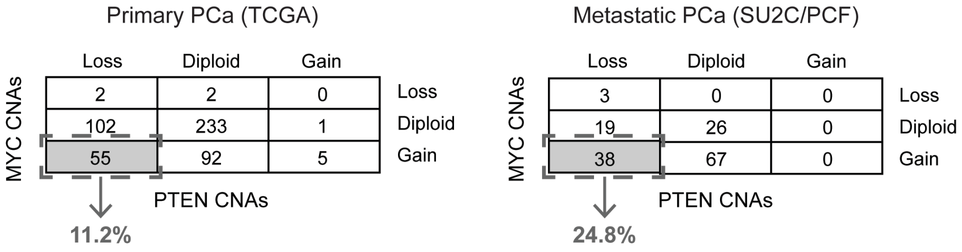


**Supplementary Figure 11. Concurrent *MYC* copy number gain and *PTEN* copy number loss represents advanced human prostate cancer.** Tables of the number of samples with *MYC* and *PTEN* copy number diploid, loss, or gain in the TCGA and SU2C/PCF databases, with gray numbers indicating the percent of samples with concurrent *MYC* gain and *PTEN* loss.

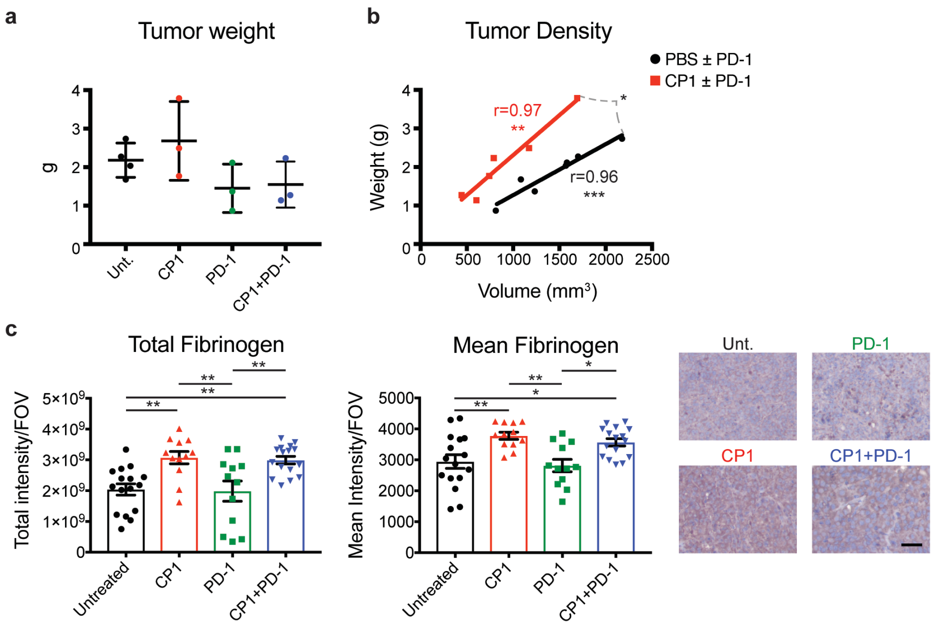


**Supplementary Figure 12. CP1 increases tumor density via fibrinous exudate.** Myc-CaP PTEN KO tumor (**a**) weights, *n* = 3-4 mice/experimental group and (**b**) densities, *n* = 6-7 mice/experimental group. (**c**) Fibrinogen IHC quantified by total and mean positivity/FOV and representative images, quadruplicate FOVs scored per sample, scale bar: 50μm, *n* = 4-6 mice/experimental group. Data represented as mean ± S.E.M. or linear regression trend lines**.** Statistical significance was determined by (**a**) two-tailed Student's *t*-test, (**b**) Pearson's correlation coefficient (r) an ANCOVA comparing slopes of trend lines, (**c**) one-way ANOVA. * $P<0.05$, ** $P<0.01$, *** $P<0.001$.

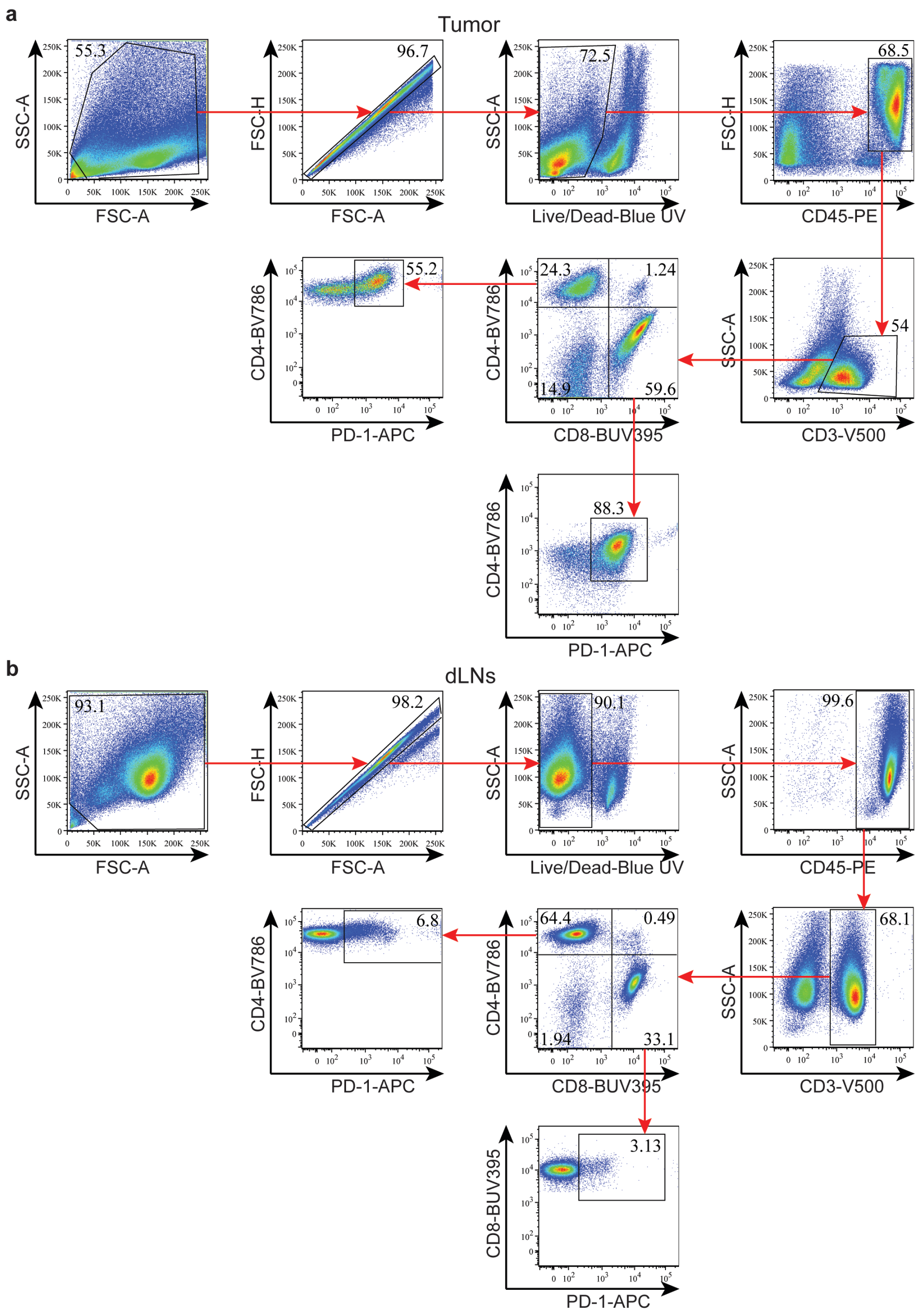


**Supplementary Figure 13. Representative flow cytometry gating strategy.** For all flow cytometry analyses, initial gating was performed on overall morphology, singlets, live cells, and CD45$^{+}$ cells, followed by antigens of interest, from (**a**) tumors or (**b**) dLNs. SSC-A = Side Scatter-Area, FSC-A = Forward Scatter-Area, FSC-H = Forward Scatter-Height.

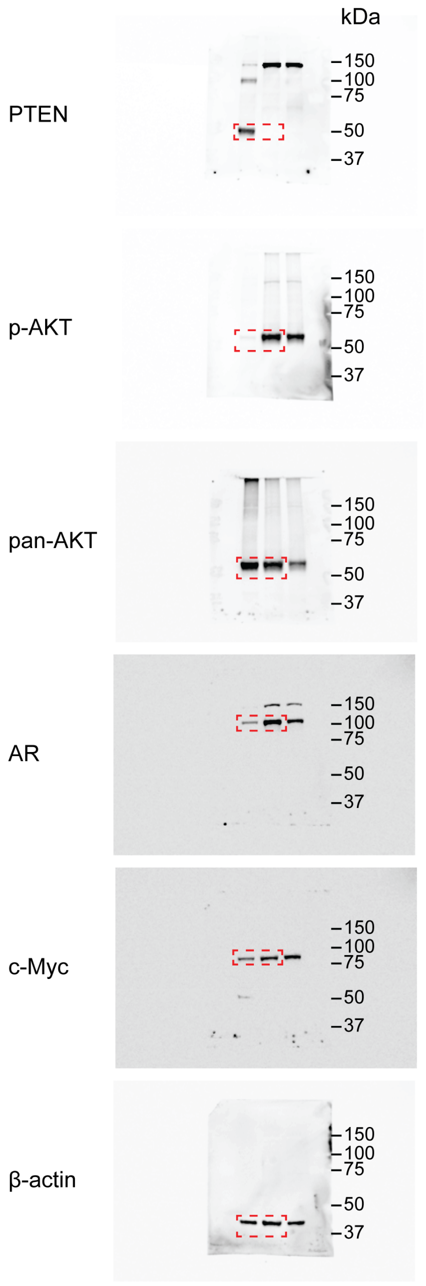


**Supplementary Figure 14. Uncropped images of western blots from Figure 5a.** Lane 1 is Myc-CaP, lane 2 is Myc-CaP PTEN KO, lane 3 is a second Myc-CaP PTEN KO clone (not included in Fig. 5a or in subsequent experiments). Red boxes represent the cropped images displayed in Fig. 5a.

**Supplementary Table 1. Primary antibodies used in this study for flow cytometry.**

| Antigen (mouse) | Label | Clone | Vendor | Catalog # |
|---|---|---|---|---|
| Calreticulin | Unconjugated | | Abcam | ab2907 |
| Annexin V | FITC | | eBioscience | 11-8005 |
| PI | - | | eBioscience | 00-6690 |
| CD45 | PE | 30-F11 | BD | 553081 |
| CD3ε | V500 | 500A2 | BD | 560771 |
| CD4 | BV786 | RM4-5 | BD | 563727 |
| CD8α | BUV395 | 53-6.7 | BD | 563786 |
| CD25 | BV421 | PC61 | BD | 562606 |
| FoxP3 | eFluor 660 | FJK-16s | eBioscience | 50-5773-80 |
| CD11b | Alexa Fluor 700 | M1/70 | BD | 557960 |
| Gr-1 | BUV395 | RB6-8C5 | BD | 563849 |
| γδ TCR | BV421 | GL3 | BD | 562892 |
| NKp46 | Alexa Fluor 700 | 29A1.4 | BD | 561169 |
| B220 | BV786 | RA3-6B2 | BD | 563894 |
| F4/80 | BV421 | T45-2342 | BD | 565411 |
| CD11c | BV786 | HL3 | BD | 563735 |
| CD80 | FITC | 16-10A1 | BD | 563727 |
| CD107a | BV786 | 1D4B | BD | 564349 |
| IFNγ | Alexa Fluor 488 | XMG1.2 | BioLegend | 505813 |
| TNFα | Alexa Fluor 700 | MP6-XT22 | BD | 558000 |
| IL-17A | BUV395 | TC11-18H10 | BD | 565246 |
| Granzyme B | eFluor 450 | NGZB | eBioscience | 48-8898-80 |
| Perforin | APC | eBioOMAK-D | eBioscience | 17-9392-80 |
| PD-1 | APC | J43 | BD | 562671 |
| PD-L1 | APC | 10F.9G2 | BioLegend | 124312 |
| PD-L2 | BV421 | TY25 | BD | 1564245 |
| CD95 | BV421 | Jo2 | BD | 562633 |
| CD95L | APC | MFL3 | eBioscience | 17-5911-80 |

**Supplementary Table 2. Primary antibodies used in this study for histology.**

| Antigen (mouse) | Dilution | Protocol | Clone | Vendor | Catalog # |
|---|---|---|---|---|---|
| *E. coli* | 1:500 | IF | | Abcam | ab20640 |
| HMGB1 | 1:1000 | IF | | Abcam | ab18256 |
| Calreticulin | 1:500 | IF | | Abcam | ab2907 |
| CD3ε | 1:100 | IHC | CD3-12 | Bio-Rad | MCA1477T |
| CD3ε | Pre-diluted | IHC | 2GV6 | Ventana | 790-4341 |
| Fibrinogen | 1:200 | IHC | ab34269 | Abcam | ab34269 |

**Supplementary Table 3. Primary antibodies used in this study for western blot.**

| **Antigen (mouse)** | **Dilution** | **Clone** | **Vendor** | **Catalog #** |
|---|---|---|---|---|
| p-MLKL | 1:1000 | EPR9515(2) | Abcam | ab196436 |
| MLKL | 1:1000 | | Abcam | ab172868 |
| RIP1 | 1:1000 | 38/RIP | BD Biosciences | 610458 |
| PARP | 1:1000 | H-250 | Santa Cruz Biotechnology | sc-7150 |
| PTEN | 1:1000 | 138G6 | Cell Signaling | 9559 |
| p-AKT | 1:1000 | S473 | Cell Signaling | 4060 |
| pan-AKT | 1:1000 | C67E7 | Cell Signaling | 4691 |
| AR | 1:2000 | N-20 | Santa Cruz Biotechnology | sc-816 |
| c-Myc | 1:1000 | Y69 | Abcam | ab32072 |
| β-actin | 1:3000 | AC-74 | Sigma | A5316 |
